# Cost-effectiveness of precision medicine: a scoping review

**DOI:** 10.1007/s00038-019-01298-x

**Published:** 2019-11-15

**Authors:** Miriam Kasztura, Aude Richard, Nefti-Eboni Bempong, Dejan Loncar, Antoine Flahault

**Affiliations:** 1 Department of Health Professions, Bern University of Applied Sciences, Bern, Switzerland; 2 Institute of Global Health, Faculty of Medicine, University of Geneva, Geneva, Switzerland

**Keywords:** Precision medicine, Economic evaluation, Scoping review

## Abstract

**Objectives:**

Precision medicine (PM) aims to improve patient outcomes by stratifying or individualizing diagnosis and treatment decisions. Previous reviews found inconclusive evidence as to the cost-effectiveness of PM. The purpose of this scoping review was to describe current research findings on the cost-effectiveness of PM and to identify characteristics of cost-effective interventions.

**Methods:**

We searched PubMed with a combination of terms related to PM and economic evaluations and included studies published between 2014 and 2017.

**Results:**

A total of 83 articles were included, of which two-thirds were published in Europe and the USA. The majority of studies concluded that the PM intervention was at least cost-effective compared to usual care. However, the willingness-to-pay thresholds varied widely. Key factors influencing cost-effectiveness included the prevalence of the genetic condition in the target population, costs of genetic testing and companion treatment and the probability of complications or mortality.

**Conclusions:**

This review may help inform decisions about reimbursement, research and development of PM interventions.

**Electronic supplementary material:**

The online version of this article (10.1007/s00038-019-01298-x) contains supplementary material, which is available to authorized users.

## Introduction

Over the last several decades, a gradual shift toward patient-centered healthcare opened the door for individualized approaches to diagnostics and treatment. Precision medicine (PM) provides “timely and cost-effective medical solutions to stratified patient subpopulations with predictable outcome margins” (Akhmetov and Bubnov 2015). The European Union’s Horizon 2020 Advisory Group defines PM as the “characterization of individuals’ phenotypes and genotypes (e.g., molecular profiling, medical imaging and lifestyle data) for tailoring the right therapeutic strategy for the right person at the right time, and/or to determine the predisposition to disease and/or to deliver timely and targeted prevention” (European Commission 2013; Nimmesgern et al. 2017).

Nowadays, PM interventions consist mostly of genetic profiling, including the detection of predictive biomarkers. These can identify patients at risk for a specific disease or a severe variant of a disease and allow for preventive interventions to reduce the burden of diseases and improve quality of life. Predictive biomarkers can also identify patients who will benefit most from certain treatments (Waldman and Terzic 2008). Furthermore, the detection of germline variations such as drug-metabolizing enzymes can help identify individuals at greater risk of adverse events or who would benefit most from dose adjustments to optimize safety (Shabaruddin et al. 2015; Waldman and Terzic 2008). Today, there are over 54,000 diagnostic tests available for over 16,400 genes (NCBI. GTR: genetic testing registry 2017). PM has the potential to reduce costs associated with inappropriate, often expensive pharmacological treatments, as well as hospitalizations for serious adverse drug reactions (Berm et al. 2016), and could ultimately allow for a more effective use of healthcare resources (Shabaruddin et al. 2015).

The cost-effectiveness of targeted interventions depends on many factors, such as the prevalence of a certain gene or allele in a population, the accuracy of a test and the costs of testing and personalized treatment (Hatz et al. 2014). As a result, patient outcomes may improve, and however, the cost-effectiveness of PM remains unclear. Recently, experts have suggested a value-based approach to PM (Patrinos and Mitropoulou 2017; Shabaruddin et al. 2015). This means measuring the value of PM interventions and demonstrating their cost-effectiveness to inform policy decisions about reimbursement and investment in research and development, particularly in solidarity-based health systems.

### Economic evaluations

Economic evaluations “identify, measure, value and compare the benefits to the costs of the alternatives being considered” (Drummond et al. 2015), both in terms of cost and outcomes, and combine them in analytical models to determine the cost per quality-adjusted life year (QALY) gained through a specific intervention compared with a standard of care (Terkola et al. 2017). In other words, a cost-effectiveness analysis evaluates if the improvement in clinical outcomes that an intervention provides is enough to justify the additional amount of money spent on it. It does not determine if an intervention reduces cost, but it tells us which intervention provides better value for the same amount of money spent (Institute of Medicine 2013). As explained in the proceedings of a workshop held by the Institute of Medicine (2013) “the best result is when outcomes improve and costs go down. The worst is when outcomes become worse and costs increase. Most (PM) interventions in healthcare result in higher costs with improved outcomes”.

Another important point to consider is the perspective adopted by different economic analyses. The models can incorporate data ranging from clinical to financial and humanistic, and include direct and indirect costs. The type of cost data included depends on the perspective adopted by the study (Lieberthal 2013). A payer perspective usually determines cost-effectiveness by comparing cost per quality-adjusted life year (QALY) gained, with a currently accepted threshold of “willingness-to-pay”. Studies adopting a societal perspective further include opportunity costs, such as out-of-pocket patient costs, other indirect medical costs, but also loss of income or productivity (Lieberthal 2013).

### Previous systematic reviews

In the past ten years, several systematic reviews of economic evaluations of PM and pharmacogenomics have been published (Berm et al. 2016; D’Andrea et al. 2015; Grosse 2015; Hatz et al. 2014; Plumpton et al. 2016; Rosso et al. 2017; Verbelen et al. 2017). Each generally examined a narrow field of PM: Verbelen or Plöthner (Plothner et al. 2016; Verbelen et al. 2017), for instance, looked at pharmacogenetic guided treatment, whereas Rosso, Grosse or Buchanan examined specific diseases or risk factors (e.g., hypercholesteremia) (Buchanan et al. 2017; Grosse 2015; Rosso et al. 2017) or adverse drug reactions in the case of Plumpton (Plumpton et al. 2016).

Most previous systematic reviews found inconclusive evidence regarding the cost-effectiveness of PM, mainly due to the insufficient quality of the studies included. The main points of criticism in this regard were inadequate sensitivity analyses, generally poor methodology, inconsistencies due to a lack of clinical evidence and low quality of data used to populate economic models, as well as heterogeneity between study designs, models and populations (Berm et al. 2016; Hatz et al. 2014; Phillips et al. 2014; Ross et al. 2012; Vegter et al. 2010). Nonetheless, a recent study reported an improvement in the quality of economic evaluations over the last few years (Shabaruddin et al. 2015). Another recommendation from previous reviews was to identify which factors influence the cost-effectiveness of PM interventions (Hatz et al. 2014). A scoping review of economic evaluations of PM could, therefore, help identify common factors and strategies for an increasingly efficient application of PM, across all fields of PM.

### Aim and research question

Our aim was to describe current research findings on the cost-effectiveness of PM and to identify characteristics of better cost-effectiveness. Specifically, we aimed to answer the following questions:

1. What is known from existing literature about the cost-effectiveness of PM?
2. Which factors influence the cost-effectiveness of PM?

## Methods

A scoping review aims to describe, summarize and facilitate dissemination of research findings. Scoping reviews provide a narrative and descriptive account of available research (Arksey and O’Malley 2005). For this scoping review, we applied the following definition of precision medicine: “clinical, therapeutic and diagnostic approaches to optimal disease management based on individual variations in a patient’s genetic profile” (U.S. National Library of Medicine 2017). PRISMA guidelines were adhered to where applicable (Moher et al. 2009).

### Search criteria

**Fig. 1:**
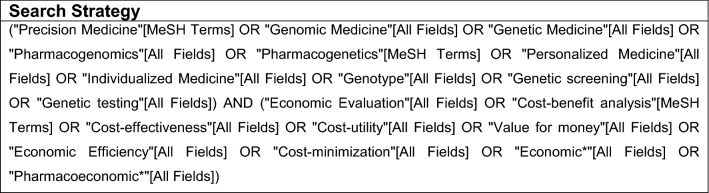
Search strategy

We used a combination of search terms based on previous reviews and aligned with the definition of PM used for this study, aiming to capture a broad range of results (Berm et al. 2016; Hatz et al. 2014; Plumpton et al. 2016; Wong et al. 2010). Keywords such as “personalized medicine”, “precision medicine” and “pharmacogenomics” were combined with search terms related to economic evaluations (Fig. 1). PubMed was chosen for the search, as it indexes the largest number of journals relevant to PM (Cesuroglu et al. 2016).

### Inclusion and exclusion criteria

In the interest of time efficiency, we only included studies published after January 2014. Several systematic reviews have been published, which include pre-2014 articles. Also, by excluding older studies, which have often been reported to lack quality (Shabaruddin et al. 2015; Wong et al. 2010), we hoped to maximize the quality of the studies included.

Further, in our initial literature search, not limited in time, it became evident that over half of the studies were published after 2014. As a scoping review aims to capture the broadest range of studies, and considering that the systematic review using the most similar range (if not the method), included studies up to 2013 (Hatz et al. 2014), we settled on studies published after January 2014.

Studies were included if they were published between January 2014 and November 2017, from a field of PM as defined for this review, evaluating economic outcomes, written in English, French or German, and full text was accessible. Articles were first screened on the title. If the title was not informative enough to form a decision with respect to these criteria, abstracts were assessed. Additional articles were identified through reference tracking.

### Data extraction

From the selected studies, the following data were extracted: (I) year of publication, (II) type disease or medical condition, (III) country, (IV) characteristics of economic analysis (type, perspective, ICER, WTP threshold applied, whenever this information was available), (V) results, (VI) conclusion of the authors, (VII) sponsorship and/or declared conflict of interest and (VII) factors influencing cost-effectiveness (see Online Resource 1). For interpretation of the outcome measure (i.e., cost-effectiveness), the conclusions as reported by the authors were used (see Online Resource 1). Two reviewers collaborated, and any disagreement was resolved in consensus.

## Results

**Fig. 2:**
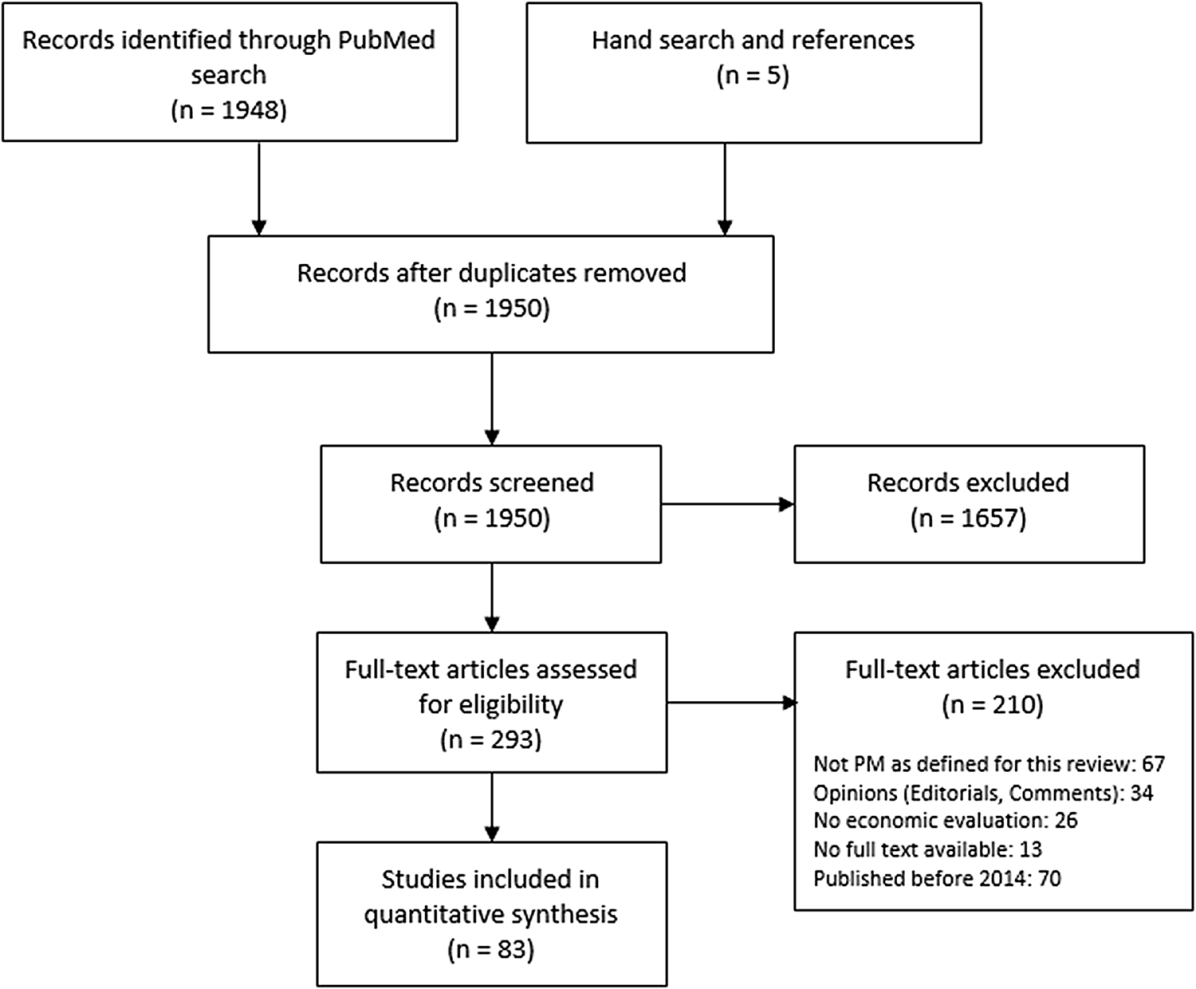
PRISMA flowchart showing the number of studies at each stage of the review process

From over 1900 results in the initial PubMed search combined with a manual reference search, a total of 83 studies were selected (see Online Resource 1 for full list). Reasons for exclusion were articles not concerned with PM as defined for this review; “opinion” articles; no economic analysis or absence of comparator; discussion of economic models/R&D only; and publication date before January 2014 and duplicates (Fig. 2).

### General characteristics

#### Geographical distribution

Since 2014, most economic evaluations of PM have been conducted in Europe and North America, with a slightly higher number in Europe (31%) (Table 1).

**Table 1:** General characteristics of included studies

Geographical distribution	*N*	% of total (*N* = 83)
Total Europe	26	31
Total Asia and Oceania	16	19
Total North America	23	28
Systematic reviews	13	16
n/a	5	6

Disease type	*N*	% of total (*N* = 83)
Cancer	36	43
Cardiovascular diseases	23	28
Adverse drug reaction	9	11
Systematic reviews covering several areas	4	5
Other (total)	11	13
Other: mental health	2	
Other: ophthalmic disease (macular degeneration)	1	
Other: autism	1	
Other: auto-immune diseases	3	
Other: MODY	2	
Other: HIV	1	
Other: asthma	1	

#### Disease type

The most frequent PM interventions included in this review related to cancer (43% of studies, *N* = 36) and cardiovascular diseases (28% of studies, *N* = 23) and adverse drug reactions (11% of studies, *N* = 9). Some other diseases were studied less frequently (13%, *N* = 11), and some systematic reviews covered several disease types (5% of studies, *N* = 4) (Table 1).

#### Genetic or pharmaceutical industry involvement

A total of 37% (*N* = 31) studies were either industry sponsored, or one or more authors were employed or financially supported by either pharmaceutical industry or industry involved in genetic testing or other areas of personalized medicine (see Online Resource 1).

### Economic characteristics

#### Type of economic analyses

Of the 83 studies included, 75% (*N* = 62) were cost-effectiveness analyses and 5% (*N* = 4) were cost-utility studies (Table 2). The remaining 20% (*N* = 17) used different methods, cost calculations or were systematic reviews.

**Table 2:** Economic characteristics of included studies

Type of analysis	*N*	% of total (*N* = 83)
Cost-effectiveness analysis	62	75
Cost-utility analysis	4	5
Other	17	20

Conclusion of authors on cost-effectiveness	*N*	% of total (*N* = 83)
Cost saving	2	2
Cost-effective	57	69
Inconclusive	9	11
Not cost-effective	14	17
n/a	1	1

Perspective of economic evaluations	*N*	% of total (*N* = 83)
Missing	11	13
n/a (SystRev or other)	16	19
Payer	20	24
Healthcare system	23	28
Societal	7	8
Healthcare system and societal	5	6
Societal and payer	1	1

#### Perspective

As seen in Table 2, around one-fifth (13%, *N* = 11) of the included studies did not provide information on the perspective chosen. Another 19% (*N* = 16) were systematic reviews or other types of review articles. The remaining studies provided information on perspective, of which 24% (*N* = 20) adopted a payer’s perspective, 28% (*N* = 23) used the perspective of the healthcare system and 8% (*N* = 7) took a societal perspective. Six percent of the studies (*N* = 5) conducted the evaluation from both a healthcare system and societal perspective, and 1% (*N* = 1) used a payer and societal perspective.

#### Cost-effectiveness: conclusion of authors

A large majority of studies (*N* = 59) conclude that the PM intervention is at least cost-effective compared to usual care (see Table 2). However, the applied willingness-to-pay thresholds vary widely, from USD 20,000/QALY, mainly in studies from the UK or Europe, to USD 200,000/QALY in studies conducted in the USA. This means that a PM intervention considered cost-effective in the USA would not necessarily fulfill the criteria to be considered so in Europe, as the amount of money per QALY, a society is willing to spend is variable.

### Factors influencing cost-effectiveness

One of the main limitations cited by authors was the insufficient quality of the data used to populate the models. Similarly, all systematic reviews report the difficulty to compare different economic analyses due to methodological inconsistencies and the heterogeneity of data included in the models. Nevertheless, similarly to others before us (Ademi et al. 2017), we noted that there were key factors that influenced the cost-effectiveness of PM. Out of all studies, 53 studies found that one or more factors influenced the cost-effectiveness of PM in their models (Table 3). The main factors, which influence cost-effectiveness, were found to be the prevalence of the genetic “condition” in the target population, costs of genetic testing and companion treatment and the probability of complications or mortality.

**Table 3:** Factors influencing cost-effectiveness

*Prevalence of “allele” in population*
Ademi et al. (2017), Alagoz et al. (2016), Barzi et al. (2015), Chen et al. (2016), D’Andrea et al. (2015, 2016), Dong et al. (2015), Gallego et al. (2015), Gonzalez et al. (2015), Grosse (2015), Ke et al. (2017), Lee et al. (2014), Moretti et al. (2017), Naylor et al. (2014), Nguyen et al. (2017), Patel et al. (2014), Plothner et al. (2016), Ruiz-Iruela et al. (2016) and Snowsill et al. (2017)
*Probability of complications (incl. mortality)*
Alagoz et al. (2016), Chong et al. (2014), Gallego et al. (2015), Gonzalez et al. (2015), Goverde et al. (2016), Jahn et al. (2015, 2017), Ke et al. (2017), Li et al. (2017), Moretti et al. (2017), Patel et al. (2014), Pink et al. (2014), Plumpton et al. (2015), Saokaew et al. (2014), Schremser et al. (2015), Snowsill et al. (2017) and You (2014)
*Cost of genetic testing*
Ademi et al. (2017), Alagoz et al. (2016), Barzi et al. (2015), Chong et al. (2014), D’Andrea et al. (2016), Dong et al. (2015), Green et al. (2014), Grosse (2015), Li et al. (2017), Martes-Martinez et al. (2017), Naylor et al. (2014), Nguyen et al. (2017), Plothner et al. (2016), Rubio-Terres et al. (2015), Snowsill et al. (2017), Wang et al. (2017) and Snowsill et al. (2015)
*Cost of companion treatment*
Buchanan et al. (2017), Gallego et al. (2015), Horster et al. (2017), Jahn et al. (2015), Lim et al. (2016), Lu et al. (2016), Narita et al. (2015), Patel et al. (2014), Ruiz-Iruela et al. (2016), Schackman et al. (2015), Yamauchi et al. (2014), You (2014, 2015) and Wallbillich et al. (2016)
*Age at testing, stage of disease*
Buchanan et al. (2017), Green et al. (2014), Jahn et al. (2015), Manchanda et al. (2015), Schremser et al. (2015) and Yamauchi et al. (2014)
*Effectiveness of test (accuracy)*
Martes-Martinez et al. (2017), Plothner et al. (2016) and Snowsill et al. (2017)
*Other* (utility/quality of life (QoL) after preventive treatment, patient adherence to treatment, uptake of genetic testing, impact of genetic testing on QoL)
Balentine et al. (2017), Green et al. (2014), Lu et al. (2016), Martes-Martinez et al. (2017), Plothner et al. (2016), Shiffman et al. (2015), Snowsill et al. (2014, 2015, 2017), Verhoef et al. (2016) and Plumpton et al. (2016)

#### Prevalence of the “allele” or “mutation” in the population tested

The prevalence of an allele of interest influences the positive predictive value of the genetic test (Ademi et al. 2017; Alagoz et al. 2016; D’Andrea et al. 2016; Gonzalez et al. 2015; Grosse 2015; Ke et al. 2017; Lee et al. 2014; Moretti et al. 2017; Naylor et al. 2014; Plothner et al. 2016; Ruiz-Iruela et al. 2016; Snowsill et al. 2017). This, therefore, increases the effectiveness of “cascade” screening programs where, for example, an individual is identified by clinical prescreening as having an increased risk of having the allele/mutation (Ademi et al. 2014; Lazaro et al. 2017).

#### Probability of complications

A higher probability of complications (including mortality) will decrease the cost-effectiveness of a PM intervention (Alagoz et al. 2016; Chong et al. 2014; Gonzalez et al. 2015; Jahn et al. 2015, 2017; Ke et al. 2017; Moretti et al. 2017; Pink et al. 2014; Plumpton et al. 2015; Saokaew et al. 2014; Schremser et al. 2015; Snowsill et al. 2017) by decreasing the number of life years gained. For the same reason, factors such as age at testing (Buchanan et al. 2017; Green et al. 2014; Jahn et al. 2015; Manchanda et al. 2015), as well as the stage of the disease (Schremser et al. 2015), will have the same effect. A person with end-stage metastatic cancer might still benefit from a PM intervention, but the cost-effectiveness ratio will be much lower, as the potential benefit is smaller. Therefore, it is more cost-effective to identify a risk or a susceptibility to a specific treatment in a younger person and/or at an earlier stage of disease.

#### Cost of genetic testing

Another factor identified in our review as a barrier to cost-effectiveness of PM interventions is the high cost of some genetic tests (Ademi et al. 2017; Alagoz et al. 2016; Chong et al. 2014; Green et al. 2014; Grosse 2015; Martes-Martinez et al. 2017; Naylor et al. 2014; Nguyen et al. 2017; Plothner et al. 2016; Rubio-Terres et al. 2015; Wang et al. 2017). However, it has been observed that the cost of genetic testing has been decreasing and is expected to continue to do so in the future (National Institute of Health NIH 2016).

The cost of treatment, if the treatment is a “companion” treatment, also influences the cost-effectiveness of PM (Buchanan et al. 2017; Horster et al. 2017; Jahn et al. 2015; Lim et al. 2016; Ruiz-Iruela et al. 2016). A companion treatment is marketed together with a specific genetic test. This is particularly relevant in oncology, where cancer “companion” treatments can have a very high cost.

#### Accuracy of the genetic test

Some studies included in our scoping review noted that the accuracy of a genetic test can influence the cost-effectiveness of PM (Martes-Martinez et al. 2017; Plothner et al. 2016; Snowsill et al. 2017). Indeed, sensitivity and specificity can play an important role in the cost-effectiveness of testing, as do the cost of false-positive or false-negative results and the unnecessary treatments and/or mortality and morbidity associated with them.

#### Other factors

Finally, factors such as the health-related quality of life (HRQoL) during and after a preventive treatment, the timeframe when a risk factor was identified, patient adherence to treatment, the uptake of genetic testing in situations of hereditary risk factors and the impact of genetic testing on HRQoL were also identified as having a potential impact on the cost-effectiveness of PM (Balentine et al. 2017; Green et al. 2014; Martes-Martinez et al. 2017; Plothner et al. 2016; Shiffman et al. 2015; Snowsill et al. 2017; Verhoef et al. 2016). Most of these factors have been previously identified, and our findings reinforce results from previous reviews (Beaulieu et al. 2010; Berm et al. 2016; Ferrusi et al. 2009; Goldie and Levin 2001; Husereau et al. 2014; Institute of Medicine 2013; Veenstra et al. 2000).

## Discussion

This study aimed to describe previous research findings on the cost-effectiveness of PM and to identify factors that influence the cost-effectiveness of PM. This scoping review described 83 studies relevant to economic evaluations and cost-effectiveness of a broad range of precision medicine interventions. Whereas most previous reviews found an overwhelming majority of studies originating from the USA (Hatz et al. 2014), we found that, since 2014, most economic evaluations of PM have been conducted in Europe and North America, with a slightly higher number in Europe (31%).

### Perspectives

In the most recent literature, there seems to be a move away from cost and toward a focus on value. Measuring the value of PM is urgently needed (Terkola et al. 2017). However, the classic economic principles—association between cost and benefits are not easily measurable in health care. Until recently, patients were not able to choose what kind of healthcare they received, nor did they pay out of their own pocket (Institute of Medicine 2013). Within privatized health insurance systems, however, patients must assume out-of-pocket costs depending on their deductible or may decide to cover a treatment that is not validated and therefore not reimbursed by their insurance. Further, patients as healthcare consumers are more aware of the options available for specific treatments or interventions, as well as potential harm and benefits of an intervention.

Some authors argue that current health economics approaches are limited, as they do not fully capture the different perspectives of value in health (Grosse et al. 2008; Institute of Medicine 2013; Terkola et al. 2017). The conversation around different financing options in healthcare, including personalized medicine, is ongoing.

Furthermore, the comparison baseline has changed. Whereas previously the comparison was between “genetic testing” and “no testing”, nowadays usual care already includes testing. For example, as Berm et al. (2016) describe KRAS testing before treatment with cetuximab in colorectal cancer, it was compared to no testing before treatment. Testing before treatment was found to be superior and was included in clinical guidelines, de facto becoming the new “usual” care. Thereafter, new treatment options will be compared to the combination of KRAS testing before cetuximab administration (Berm et al. 2016).

### Challenges for economic evaluations in precision medicine

Many challenges for economic evaluations in PM have previously been described (Faulkner et al. 2012; Hatz et al. 2014; Terkola et al. 2017). Economic models have significant limitations compared with clinical trials. Data from clinical trials, which are usually extrapolated to populate the models, may not be transferable into a real-world setting (Lieberthal 2013). Further, the increasing complexity of PM interventions, such as sequential or cascade testing, which is reflected in the models, results in a higher level of uncertainty about the incremental cost-effectiveness ratio (ICER) (Terkola et al. 2017).

#### Lack of data

There is still a lack of clinical evidence to support PM technologies, particularly of “real-world” data on clinical utility, which is not model based or derived from selective trials (Akhmetov and Bubnov 2015; Berm et al. 2016; Brüggenjürgen et al. 2012; Phillips et al. 2014; Terkola et al. 2017). There is an even larger uncertainty with pharmacogenomic tests, since they have no direct influence on patient outcomes, as they do not treat patients directly, but rather improve a clinician’s decisions about treatment (Akhmetov and Bubnov 2015).

As with clinical data, there is a lack of real-world cost data, and of data where opportunity costs are reflected (e.g., the costs for genetic counseling and the costs of ambiguous test results) (Conti et al. 2010).

Besides this, several authors have raised the issue of cost for testing inaccuracies (Conti et al. 2010; Goldie and Levin 2001; Terkola et al. 2017). It is argued that for a cost-effectiveness analysis to be useful to inform policies, it should include all clinical and economical events triggered by the test results, such as the cost of false-positive and false-negative results. In an example explained by Goldie (Goldie and Levin 2001), for a woman who tests positive for the BRCA-1 mutation but is not destined to develop breast cancer (estimated at 30%), the benefits of a prophylactic mastectomy would be negligible but she would still bear the huge costs of the psychological anxiety and healthcare resources associated with lifelong screening.

#### “Willingness-to-pay” thresholds

As our review demonstrates, there is no consensus on a willingness-to-pay threshold for a quality-adjusted life year gained. In the USA, the most commonly used willingness-to-pay threshold is 50,000 USD per QALY, historically based on the cost-effectiveness of dialysis from the 1970s, whereas currently, the cost-effectiveness ratio of dialysis is 130,000 USD per QALY (Coate and Leighl 2011). WHO recommends a willingness-to-pay threshold of 3 times the GDP of the country. This threshold, however, was only applied in 3 out of 83 studies included in this scoping review (Brown et al. 2015; Horster et al. 2017; Schremser et al. 2015). It also has been noted that QALYs lack the ability to fully capture all aspects of health outcomes (Garrison et al. 2017; Terkola et al. 2017).

### Limitations of this study

This scoping review has several limitations. First, we only searched one database, which reduces the potential breadth and depth of the results. Second, the chosen definition of PM (narrower, e.g., no lifestyle, nor prenatal diagnostics included) and search terms (searching for articles identified as “personalized” or “precision” medicine) did not include all individual genetic tests. This has the potential to limit the scope of the results.

### Conclusion

PM interventions have been increasingly useful for screening, testing and treatment of many diseases. Due to the many factors which influence cost-effectiveness and the varied thresholds of willingness-to-pay applied, the cost-effectiveness of PM remains unclear. Therefore, we might require a different approach to value precision medicine interventions.

## Electronic supplementary material

Below is the link to the electronic supplementary material.

Supplementary material 1 (PDF 132 kb)
