# Supplementary material for: Cost-effectiveness of precision medicine: a scoping review
Source: Int J Public Health. 2019 Nov 15;64(9):1261–71. doi: 10.1007/s00038-019-01298-x (PMC6867980; doi:10.1007/s00038-019-01298-x)
Supplement: Supplementary file 1 — Supplementary material 1 (PDF 132 kb) [file 38_2019_1298_MOESM1_ESM.pdf]

ESM_1 - Supplementary Material

1. **Summary table of included studies**

1. Summary table of included studies

Authors	Year	Country	Medical condition / Disease type	Type of economic evaluation	Perspective	Authors' conclusion on cost-effectiveness	ICER*	Willingness -to-pay threshold	Sponsorship / Funding / Potential conflict of interest	Factors influencing cost-effectiveness
Ademi Z,et al.	2017	SystRev	Cardiovascular disease	other	missing	inconclusive	n/a		Public and Industry	Positive Predictive Value (PPV), Cost of testing
Ademi Z, et al.	2014	Australia	Cardiovascular disease	CEA	healthcare system	yes	AU$3'565/QALY	not given	Public Funding	none mentioned
Alagoz O, et al.	2016	USA	Adverse Drug Reaction	CEA	missing	yes	$43'165 per additional LY and $53'680 per additional QALY	$100'000 per QALY	Industry	Complication/Mortality, PPV, Cost of testing
Alberts SR, et al.	2014	USA	Cancer	other	payer	yes	$US991 per 0.114 QALYs	not given - cost comparison only	Industry	none mentioned
Balentine, CJ, et al.	2017	other	Cancer	CEA	missing	no	$9'400 per 4.47 quality-adjusted life-years vs $6'100 per 4.50 QALYs for the standard procedure. There was only a 0.3% probability of PM being cost saving and a 14.9% probability of improving QALYs	$100'000/ QALY	Public and Industry	Other
Barzi A, et al.	2015	USA	Cancer	CEA	missing	no	$35'143/LYG	$50'000/LYG	Public	Costs of testing, PPV
Bentley TG, et al.	2014	USA	Cancer	CEA	payer	yes	$50'273/QALY	$100'000/ QALY	Industry	none mentioned
Berm EJ, et al.	2016	SystRev	SystRev	other	n/a	yes	N/A	N/A	Industry	none mentioned
Borse MS, et al.	2017	USA	Cardiovascular disease	CEA	payer	yes	$42'198 per major event avoided at one-year post intervention	n/a	none declared.	none mentioned
Brown GC et al.	2015	USA	Other	CUA	societal and payer	yes	net societal cost saving of $160'582 per early treatment patient	3xGDP	Industry	none mentioned
Brown LC, et al.	2017	USA	Other	other	n/a	yes	medication cost saving of $3'988/patient/year	n/a	Industry	none mentioned

Buchanan J, et al.	2017	UK	Cancer	other	healthcare system and societal	no	n/a (no ICER)	£30'000/QALY or LYG	none declared	Cost of treatment; Age at testing/Stage of disease
Chen YE, et al.	2016	Taiwan	Cancer	CEA	healthcare system	yes	$6’025 for Strategy 1 to $145’110 for Strategy 4, whereas ICERs with respect to the next most cost-effective strategy ranged from $6’025 for Strategy 1 to $988’217 for Strategy 4	$50'000/QALY	Public	PPV, uptake of screening
Chong HY, et al.	2017	Malaysa	Adverse Drug Reaction	CEA	societal	no	LOSS of 0.0255 QALYs at an additional cost of $707; LOSS of 0.2622 QALYs at an additional cost of $4’127	For universal screening: MYR 37 000 ($8982)/QALY	Public funding	Cost of testing,
Chong HY, et al	2014	Thailand	Cardiovascular disease	CEA	healthcare system and societal	no	1’477’042 THB (49’234 USD) per QALY or 1’473’852 THB [49’128 USD] per QALY	less than 1.2 times the per-capita GNI (160,000 THB = $5’333)	none declared	Cost of testing
Cortejoso L, et al.	2016	Spain	Cancer	CEA	n/a	yes	n/a	if min 2.1 cases of severe event per 1000 get prevented	none declared.	none mentioned
Cromwell I, et al.	2016	Canada	Cancer	CEA	missing	yes	CAN$8’123/0.64 QALY and 0.18 LYG	not given	Public	None mentioned
D'Andrea E, et al,	2016	SystRev	Cancer	other	n/a	inconclusive	n/a	$50,000–100,000/QALY or LYG for USA, and £20,000–30,000/QALY or LYG for UK	Public	PPV, Cost of testing

D'Andrea E, et al.	2015	SystRev	Cancer	other	n/a	yes	n/a	€37,000/QALY	Public	none mentioned
Dong D, et al.	2015	Singapore	Adverse Drug Reaction	CEA	healthcare system	no	$85'630/QALY and LOSS of 13'510USD/QALY (reduced QALYs)	$50'000/QALY	none declared	PPV, Cost of testing
Eccleston A, et al.	2017	UK	Cancer	CEA	healthcare system	yes	£4'339/QALY	£20'000/QALY	Industry and Public	none mentioned
Elias J, et al.	2015	Netherlands	Other	other	n/a	no	n/a	not given - cost comparison only	none declared.	none mentioned
Fischer L, et al.	2015	SystRev	Cancer	CEA	n/a	yes	ranged from cost-savings of €3'548 per patient to additional costs of €9'113/QALY	not given	non declared	none mentioned
Gallego CJ, et al.	2015	USA	Cancer	CEA	societal	yes	$36,500/QALY	$100'000/QALY	Public	PPV, Cost of treatment
Garrison LP Jr, et al.	2015	USA	Cancer	other	missing	n/a	n/a	n/a	Industry	none mentioned
Gonzalez FM, et al.	2015	SystRev	Cardiovascular disease	CEA	n/a	yes	n/a	$50'000/QALY	None declared.	PPV, Complications/Mortality
Goverde A, et al.	2016	Netherlands	Cancer	CEA	missing	yes	Max. €13'000/LYG	€30'000/QALY, €40'000/LYG	Public	Complications/Mortality
Green LE, et al.	2014	USA	Cancer	CEA	payer	yes	Max. US$65'000/QALY	$100'000/QALY	Industry	Cost of testing, age at testing/stage of disease, other
Grosse SD.	2015	SystRev	Cancer	CEA	n/a	yes	Max. $100'000/LYG or QALY, in all but one study	$100'000/QALY	none declared	PPV, Cost of testing
Hatz MH, et al.	2014	SystRev	SystRev	CEA	n/a	inconclusive		n/a	Public	none mentioned
Hochheiser LI, et al.	2014	USA	Cardiovascular disease	other	payer	yes	cost savings of $0.77/person/month	not given	Industry	none mentioned
Hörster L, et al.	2017	Germany	Cancer	CEA	missing	yes	$59'136/LYG	3xGDP	Public	Cost of treatment

Jahn B, et al.	2015	Austria	Cancer	CEA	societal	yes	€15’700/QALY	$65’914 or $25’435 /QALY	Public	Complications/Mortality, Cost of treatment, Age at testing/Stage of disease
Jahn B, et al.	2017	other	Cancer	CEA	healthcare system	yes	multiple	€50’000 or €100’000/ QALY	Public	Complications/Mortality
Jiang M, et al.	2016	Hong Kong	Cardiovascular disease	CEA	healthcare system	yes	$75’208 cost and 7.6249QALY gained	$50'000/QALY	none declared.	none mentioned
Johnson SG, et al.	2015	USA	Cardiovascular disease	other	payer	yes	annual savings $444,852 per 1000 patients	not given - cost comparison only	Industry	none mentioned
Ke CH, et al.	2017	Taiwan	Adverse Drug Reaction	CEA	payer	yes	New Taiwan NT 234’610 (=$7’508)/QALY	3xGDP $22’635 to $67’905	Public	PPV, Complications/Mortality
Kim DJ, et al.	2017	Korea	Cardiovascular disease	CEA	healthcare system	yes	$1356.2/QALY	$50'000/QALY	Public	none mentioned
Làzaro P, et al.	2017	Spain	Cardiovascular disease	CEA	healthcare system and societal	yes	€29’608 per QALY	€30’000/QALY	Public	none mentioned
Lee WS, et al.	2014	USA	Cancer	CUA	healthcare system and societal	no	$33.96/QALY	cost comparison	nothing mentioned	PPV
Li Y, et al.	2017	USA	Cancer	CEA	payer	yes	$42,067/LYG or $69,920/QALY for the 50-year-old cohort and $23,734/LYG or $48,328/QALY for the 40-year-old cohort.	$100'000/ QALY	Industry	Complications/Mortality, Cost of treatment, Cost of testing
Lim EA, et al.	2016	Korea	Cancer	CEA	payer	yes	the average medical costs were $23,952 USD in the no-testing strategy and $23,334 USD in the testing strategy, and QALYs gained were 0.556 and 0.635 respectively.	not given	Public	Cost of treatment
Lobo JM, et al.	2017	USA	Cancer	CEA	missing	yes	$90’833/QALY	$100'000/ QALY	Industry	none mentioned

Lu S, et al.	2016	China	Cancer	CEA	healthcare system	inconclusive	$16’820, $16’850 and $24’424/QALY for the 3 testing strategies when 1) PAP available, 2) no PAP available, 3) more than $220,000/QALY	$32’000/QALY	Industry	Cost of treatment (model only cost-effective if government patient assistance program to finance treatment is included into model), other
Luime JJ, et al.	2015	Netherlands	Other	CEA	societal	yes	Max ICER €5’314	€20’000/QALY	none declared.	none mentioned
Manchanda R, et al.	2014	UK	Cancer	CEA	missing	yes	-£2079/QALY	£20'000/QALY	Industry	Age at testing/stage of disease
Manchanda R, et al.	2017	UK	Cancer	CEA	payer	yes	“Population testing for BRCA mutations is cost-saving in Ashkenazi-Jewish women with 2, 3, or 4 grandparents (22-33 days life gained) in the United Kingdom and 1, 2, 3, or 4 grandparents (12-26 days life-gained) in the United States populations, respectively. It is also extremely cost-effective in women in the United Kingdom with just 1 Ashkenazi-Jewish grandparent	£20'000 - £30'000/QALY and £100'000/QALY	Public	none mentioned
Martes-Martinez C, et al.	2017	USA	Cardiovascular disease	CUA	healthcare system	no	ICUR (cost-utility) $127’501/QALY	$50'000/QALY	none declared.	Cost of testing, accuracy of test, other
Mitropoulou C, et al.	2015	Croatia	Cardiovascular disease	CEA	payer	yes	€31’225/QALY	not given	Public and industry	none mentioned
Mitropoulou C, et al.	2016	Serbia	Cardiovascular disease	CEA	payer	cost-saving	cost-saving	not given	Public and industry	none mentioned
Moretti ME, et al.	2017	Canada	Adverse Drug Reaction	CEA	healthcare system and societal	yes	CAN$10’433/QALY	not given	Public	PPV, Complications/Mortality,
Narita Y, et al.	2015	Japan	Cancer	CEA	payer	yes	¥3.38 million ($32,500)/QALY	¥5 million ($50'000)	Industry	Cost of treatment

Naylor, RN, et al.	2014	other	other	CEA	healthcare system	no	$205’000/QALY	From $50’000/QALY to $109’000–$297’000/QALY	Public	PPV, Cost of testing
Nguyen HV, et al.	2017	Singapore	other	CEA	payer	no	$93’663/QALY	$50'000/QALY	Public	PPV, Cost of testing
Patel V, et al.	2014	USA	Cardiovascular disease	CUA	healthcare system	yes	ICUR $4’200	$100'000/QALY	Industry	Complications/Mortality, PPV, Cost of treatment
Pink J, et al.	2014	other	Cardiovascular disease	CEA	healthcare system	yes	£13’226/QALY to £19,858/QALY depending on strategy.	comparison	Public	Complications/Mortality
Plöthner M, et al.	2016	Germany	SystRev	CEA	n/a	inconclusive	n/a	influencing factors	None declared	PPV, Cost of testing, Accuracy of test, Other
Plumpton CO, et al.	2015	UK	Adverse Drug Reaction	CEA	healthcare system	yes	£12’808/QALY	£20'000/QALY	Public	Complications/Mortality
Plumpton CO, et al.	2016	SystRev	Adverse Drug Reaction	other	n/a	yes	n/a	n/a	Public	Other (needs clinical evidence)
Rosenblat JD, et al.	2017	SystRev	other	CEA	n/a	inconclusive	n/a	$50’000/QALY	Industry	none mentioned
Rosso A, et al.	2017	SystRev	Cardiovascular disease	CEA	n/a	yes	n/a	£20’000–£30’000/QALY or LYG for UK; $50’000–$100,000/QALY or LYG in USA	Public	none mentioned
Rubio-Terrés C, et al.	2015	Spain	Cardiovascular disease	other	healthcare system	yes	n/a	€30'000	Industry	Cost of testing
Ruiz-Iruela C, et al.	2016	Europe	Adverse Drug Reaction	CEA	missing	yes	€306 per event avoided	not given - cost comparison only	none declared	PPV, Cost of treatment

Saokaew S, et al.	2014	Thailand	Adverse Drug Reaction	CEA	societal	yes	$5’062/QALY	1.2 times Gross National Income (GNI) per capita or 160,000 THB (USD 5,161)	Public	Cost of testing, cost of treatment, Complications/Mortality
Schackman BR, et al.	2015	USA	other	CEA	healthcare system	yes	non genetic strategy >$100'000/QALY	$100'000/ QALY	Public and Industry	Cost of treatment
Schremser K, et al.	2015	Germany	Cancer	CEA	payer	yes	€15’577/QALY	3xGDP €70’500–€106’000 in Germany or £20’000–£30000/QALY in UK	Public and Industry	Complications/Mortality, Age at testing/stage of disease
Segui MA, et al.	2014	Spain	Cancer	CEA	healthcare system	yes	€6’169/QALY at 10 years, €287/QALY at lifetime	€30’000/LYG and QALY	none declared	none mentioned
Severin F, et al.	2015	Germany	Cancer	CEA	payer	no	ranges between €77’268 per LYG in strategy B-2 and €4’188’036 per LYG in strategy 7	threshold of €50’000/LYG	Industry	None mentioned
Shiffman D, et al.	2015	USA	cardiovascular disease	CEA	payer	yes	$47’148) if 2.1 % or more of the test positive patients were to adhere to warfarin	not given	Industry	Other (adherence)
Snowsill T, et al.	2014	UK	Cancer	CEA	healthcare system	yes	£5491/QALY for strategy 5 to £9571/QALY for strategy 8	£20'000/QALY	Public	PPV, other
Snowsill T, et al.	2017	UK	Cancer	CEA	healthcare system	yes	£11’008/QALY	£20,000/QALY	Public	PPV, Complications/mortality, other
Snowsill T, et al.	2015	UK	Cancer	CUA	healthcare system	yes	£5’491/QALY	£20'000/QALY	Public	Cost of testing, other (utility)
Thompson AJ, et al.	2014	UK	other	CEA	healthcare system	inconclusive	£256.89/QALY	£20'000/QALY	Public	none mentioned
Verbelen M, et al.	2017	SystRev	SystRev	other	n/a	yes	n/a	different	Public and industry	none mentioned

Verhoef TI, et al.	2015	Netherlands	Cardiovascular disease	CEA	healthcare system	no	€28’349 and €24’427 for different strategies	€20'000/QALY	Public and industry	none mentioned
Verhoef TI, et al.	2016	Sweden	Cardiovascular disease	CEA	healthcare system	yes	£6’702/QALY in the UK and 253’848 SEK in Sweden	£20'000/QALY and SEK500'000/QALY (= £40’000)	Public and Industry	Other
Wallbillich JJ, et al.	2016	USA	Cancer	CUA	payer	no	$479’303/QALY	$100'000/QALY	none declared.	Cost of treatment
Wang Y, et al.	2017	Hong Kong	Cardiovascular disease	CEA	healthcare system	yes	$2’560/QALY	< 1xGDP = $42’423/QALY	none declared	Cost of testing
Wu AC, et al.	2015	USA	other	other	societal	cost-saving	cost-saving	not given - cost comparison only	Public	none mentioned
Yamauchi H, et al.	2014	Japan	Cancer	CEA	societal	yes	$6368/QALY or ¥636’752/QALY	¥6 million/QALY	Industry	Cost of treatment, Age at testing/Stage of disease
You JH.	2015	Hong Kong	Cardiovascular disease	CEA	payer	yes	cost-saving but reduces QALY. Not using genotype-guided DOAC resulted in ICER of $314’129/QALY	$50’000/QALY	Public	Cost of treatment
You, JH.	2014	other	Cardiovascular disease	CEA	payer	yes	$2843/QALY	$50'000/QALY	Public	Complications/Mortality, Cost of treatment
Ziegler A, et al.	2017	SystRev	other	other	n/a	inconclusive	n/a	n/a	none declared	none mentioned

* incremental cost-effectiveness ratio: amount of money per QALY or LYG (Year of life gained) compared to std)
